# The Interplay between Transcription Factor SALL4 and Histone Modifiers in Hematopoietic Stem and Progenitor Cells

**DOI:** 10.33696/immunology.3.073

**Published:** 2021

**Authors:** Hiro Tatetsu, Daniel G. Tenen, Li Chai

**Affiliations:** 1 Department of Hematology, Rheumatology and Infectious Diseases, Kumamoto University Hospital, Kumamoto, Japan, 860-8556; 2 Cancer Science Institute of Singapore, National University of Singapore, Centre for Translational Medicine (MD6), #12-01, 14 Medical Drive, Singapore 117599; 3 Harvard Stem Cell Institute, Center for Life Science Room 437, 3 Blackfan Circle Room 437, Boston, MA 02115, USA; 4 Department of Pathology, Brigham and Women’s Hospital, Harvard Medical School, 75 Francis Street, Boston, MA 02115, USA

## Commentary

Currently, there is a growing need for culturing hematopoietic stem/progenitor cells (HSPCs) *ex vivo* for various clinical applications such as HSPC transplantation and gene therapy. For many patients with hematologic, genetic, and immune diseases, HSPC transplants can be a life-saving treatment. There are over 20,000 patients in the US receiving HSPC transplantation yearly [1]. About two-thirds of these cases are autologous and the rest are allogeneic transplants. The sources of the HSPCs are from peripheral blood mobilized stem/progenitor cells (PBSC), cord blood (CB) and bone marrow (BM). Umbilical cord blood can be an excellent HSPC donor source; however, its use is severely constrained by the limited HSPC numbers in one single cord blood unit. Developing technologies that allow *ex vivo* expansion of cord blood will be highly beneficial for the clinical application of HSPC transplants. In addition, there is a growing need for culturing PBSC *in vitro* for transplant-related applications such as gene therapy or genome-editing via TALENs or CRISPR/Cas9 [2,3]. Furthermore, the same PBSC *in vitro* culture technique can be used for HSCP expansion for poor autologous mobilizations to avoid additional collections. Establishing culture conditions that can maintain and expand HSPCs from PBSC *ex vivo* will be beneficial to these clinical applications.

Expansion of HSPCs in culture, in general, is at the expense of “stemness”. Since the fate of a HSPC is governed by genetic and epigenetic factors, and we and others have proposed that the fate of a HSPC can be redirected or reprogrammed through modifications of key factors. Many efforts have been applied to identify and understand the key players in HSPC cell fate switching process through celltype-specific gene expression profile, cellular surface markers and functional studies. Genes and pathways that are functionally linked to self-renewal of HSCs include CEBPα [4], Notch ligands [5,6], Angiopoietin-like proteins [7], SALL4 [8], homeobox protein B4 (HOXB4) [9], c-MPL [10], as well as various methods in HSCP expansion, such as the use of Prostaglandin E2 [11,12], Pleiotrophin [13], SR1 [14], UNC0638 [15], Pyrimidoindole derivatives [16], and TEPA [17,18] have been reported.

We have searched for a robust and short-term *ex vivo* culture condition that can maintain and expand PBSCs without the loss of their “stemness”. We utilized a 3- to 5-day assay that can be easily modified to use in the current clinical HSPC transplantation setting, and co-expression of CD34 and CD90 to identify compounds with potentials for PBSC expansion [19]. After surveyed 466 compounds, including multiple epigenetic modifiers, we have identified ten compounds to be positive hits based on the increased percentage of CD34^+^CD90^+^ cells. Five compounds were histone deacetylase inhibitors (HDACis), and they are trichostatin A (TSA), DLS3, valproic acid (VPA), vorinostat also known as suberanilohydroxamic acid (SAHA), and Merk60. Especially, treatment of CD34^+^ PBSCs with a single dose of TSA ex vivo yielded the greatest expansion (11.7-fold) of CD34^+^CD90^+^ cells when compared to the control group. Additionally, TSA-treated PBSC CD34^+^ cells had a statistically significant higher engraftment rate than the control-treated group in xenotransplantation experiments.

**Figure 1:**
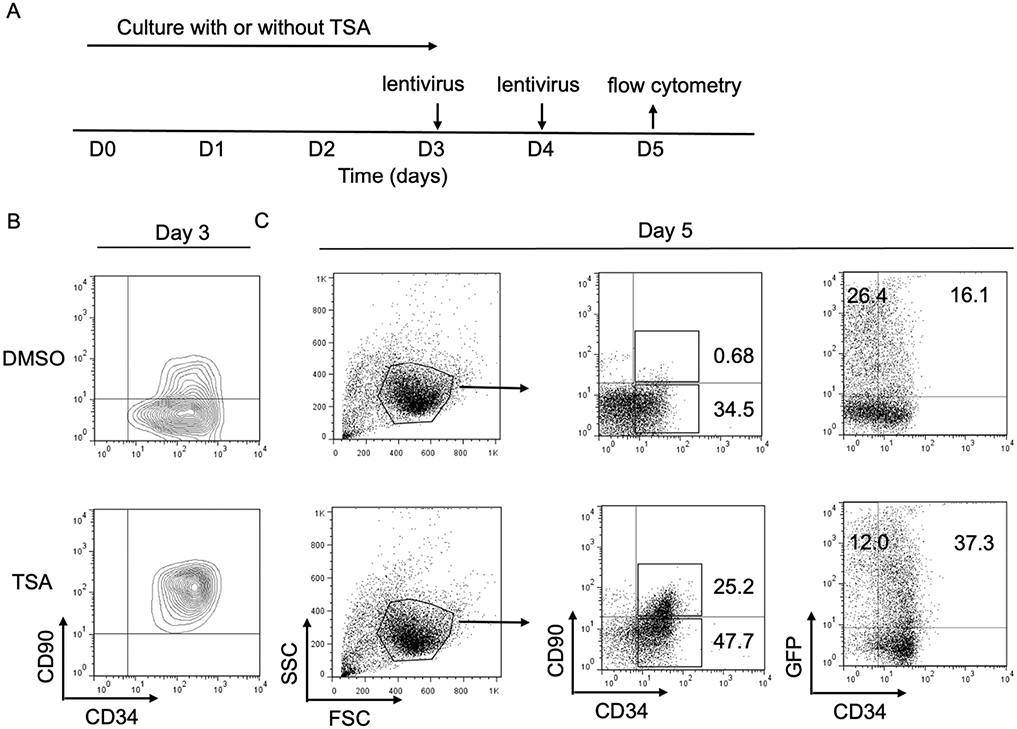
Treatment of PBSC CD34^+^ cells with TSA increased the efficiency of gene insertion approach. **(A)** Cells were cultured with TSA for 72 hours, then cells were infected in medium containing lentiviral particles containing green fluorescent protein (GFP). The culture medium was then removed and replaced with fresh media. **(B)** CD34 and CD90 expression with or without TSA treatment on day 3. **(C)** CD34^+^CD90^+^cells and CD34^+^GFP^+^cells with or without TSA treatment on day 5.

Since cultured CD34^+^CD90^+^ cells could maintain stem/progenitor cell properties based on our *in vitro* and *in vivo* experiment results, we next asked whether the expansion of CD34^+^CD90^+^ cells by TSA could affect the lentiviral transduction efficacy during *ex vivo* culture. A transfer vector encoding a green fluorescent protein (GFP) was used for the evaluation of lentiviral transduction. PBSC CD34^+^ cells were cultured with or without TSA for 3 days, followed by lentiviral transduction with protamin twice for 48 hours (Figure 1A). On day 3, we noticed that the majority of TSA treated cells were CD34^+^CD90^+^ cells (Figure 1B). In addition, we observed that the percentage of CD34^+^CD90^+^ cells, as well as CD34^+^GFP^+^ cells treated with TSA, was higher than that of cells without treatment on day 5 (Figure 1C). These data suggested that our *ex vivo* expansion method could promote lentiviral transduction for CD34^+^CD90^+^ cells *ex vivo*.

We are intrigued by the finding that HDACi is enriched as a class of molecules that can be used to expand HSPCs *ex vivo*. Since acetylation of histone tails can regulate the accessibility of transcription factors to DNA, increased acetylation is generally associated with an “open”, active state of chromatin, while hypo-acetylation is characteristic of a repressive chromatin state. This post-translational modification of histone tail is regulated by the opposing activities of histone acetyltransferase (HAT) and histone deacetylase (HDAC) enzymes. The HDACi treatments of cells in general lead to a more open chromatin and gene activation. We propose that since the HSPC fate is governed by key transcription factors, which are downregulated during HSPC *ex vivo* expansion; by adding HDACi to the culture medium, we can keep the chromatin in a relative “open” state, and maintain the expression of these key factors, therefore, maintain the “stemness” of the expanded HSPCs. We further demonstrate that among multiple key regulators involving in the self-renewal property of HSPCs, SALL4 is important for this TSA-mediated *ex vivo* CD34^+^CD90^+^ HSPC expansion process [19].

SALL4 was originally cloned based on its DNA sequence homology to the homeotic gene in Drosophila, *spalt* (*sal*) [20-23]. Several pieces of evidence support that SALL4 plays an essential role in maintaining the pluripotent and self-renewal properties of embryonic stem cells (ESCs). In adult mice, Sall4 expression is mostly restricted to germ cells, wherein it is highly expressed in undifferentiated spermatogonia and oocytes in primordial, primary, and secondary follicles [24-26]. Similarly, the expression of SALL4 in adult human tissue is restricted to the testis and ovary [27]. One exception to this expression pattern is human CD34^+^ (HSPCs) [28]. It has been reported that overexpression of SALL4 in mobilized peripheral blood CD34^+^ cells increased *ex vivo* expansion efficiency by more than 10,000 fold for CD34^+^/CD38^−^ and CD34^+^/CD38^+^ with appropriate cytokines [8]. Similarly, Shen et al. demonstrated SALL4B isoform expanded BM-derived CD34^+^ non-human primate HSCs [29] and Mossahebi-Mohammadi M. et al demonstrated that SALL4 lentiviral transduction causes a 6-fold change in total cell count CD133^+^HSCs compared to the control group [30]. In addition, Milanovich et al. mentioned SALL4 dose may important for functioning in murine hematopoiesis [31]. These studies indicate SALL4 is one of the key factors for the maintenance and enhancement of HSPCs.

Further studies are needed to understand the mechanism(s) SALL4 function in HSPCs and to apply for the clinical setting. The mechanism(s) of SALL4 in gene regulation, at least in part, is through its interaction with epigenetic complexes (also known as chromatin modifiers). SALL4 can function as a gene repressor and activator through its interactions with both gene-activating and repressing epigenetic complexes. More specifically, SALL4 can repress its target genes through its interaction with the Nucleosome Remodeling and Deacetylase complex (NuRD) [32-35], an HDAC-containing epigenetic repressor complex. It can also activate its target gene *HOXA9* through its interaction with Mixed-Lineage Leukemia (MLL) protein [36], which is the key member of an epigenetic activator complex. It remains to be determined whether both the activation and repression functions of SALL4 are required for HSPC expansion, and the development of novel pharmacological tools to modulate these pathways could lead to new HSPC expansion technology.

## References

[1] D’Souza A, Fretham C, Lee SJ, Arora M, Brunner J, Chhabra S, Current Use of and Trends in Hematopoietic Cell Transplantation in the United States. Biol Blood Marrow Transplant. 2020;26(8):e177–e82. 32438042 10.1016/j.bbmt.2020.04.013 PMC7404814

[2] Carlson DF, Tan W, Lillico SG, Stverakova D, Proudfoot C, Christian M, Efficient TALEN-mediated gene knockout in livestock. Proc Natl Acad Sci U S A. 2012;109(43):17382–7. 23027955 10.1073/pnas.1211446109 PMC3491456

[3] Hsu PD, Lander ES, and Zhang F. Development and applications of CRISPR-Cas9 for genome engineering. Cell. 2014;157(6):1262–78. 24906146 10.1016/j.cell.2014.05.010 PMC4343198

[4] Zhang P, Iwasaki-Arai J, Iwasaki H, Fenyus ML, Dayaram T, Owens BM, Enhancement of hematopoietic stem cell repopulating capacity and self-renewal in the absence of the transcription factor C/EBP alpha. Immunity. 2004;21(6):853–63. 15589173 10.1016/j.immuni.2004.11.006

[5] Delaney C, Varnum-Finney B, Aoyama K, Brashem-Stein C, and Bernstein ID. Dose-dependent effects of the Notch ligand Delta1 on ex vivo differentiation and in vivo marrow repopulating ability of cord blood cells. Blood. 2005;106(8):2693–9. 15976178 10.1182/blood-2005-03-1131 PMC1366491

[6] Delaney C, Heimfeld S, Brashem-Stein C, Voorhies H, Manger RL, and Bernstein ID. Notch-mediated expansion of human cord blood progenitor cells capable of rapid myeloid reconstitution. Nat Med. 2010;16(2):232–6. 20081862 10.1038/nm.2080 PMC2819359

[7] Zhang CC, Kaba M, Ge G, Xie K, Tong W, Hug C, Angiopoietin-like proteins stimulate ex vivo expansion of hematopoietic stem cells. Nat Med. 2006;12(2):240–5. 16429146 10.1038/nm1342 PMC2771412

[8] Aguila JR, Liao W, Yang J, Avila C, Hagag N, Senzel L SALL4 is a robust stimulator for the expansion of hematopoietic stem cells. Blood. 2011;118(3):576–85. 21602528 10.1182/blood-2011-01-333641 PMC3142902

[9] Amsellem S, Pflumio F, Bardinet D, Izac B, Charneau P, Romeo PH, Ex vivo expansion of human hematopoietic stem cells by direct delivery of the HOXB4 homeoprotein. Nat Med. 2003;9(11):1423–7. 14578882 10.1038/nm953

[10] Nishino T, Miyaji K, Ishiwata N, Arai K, Yui M, Asai Y, Ex vivo expansion of human hematopoietic stem cells by a small-molecule agonist of c-MPL. Exp Hematol. 2009;37(11):1364–77e4. 19744539 10.1016/j.exphem.2009.09.001

[11] Goessling W, Allen RS, Guan X, Jin P, Uchida N, Dovey M, Prostaglandin E2 enhances human cord blood stem cell xenotransplants and shows long-term safety in preclinical nonhuman primate transplant models. Cell Stem Cell. 2011;8(4):445–58. 21474107 10.1016/j.stem.2011.02.003 PMC3148081

[12] Cutler C, Multani P, Robbins D, Kim HT, Le T, Hoggatt J, Prostaglandin-modulated umbilical cord blood hematopoietic stem cell transplantation. Blood. 2013;122(17):3074–81. 23996087 10.1182/blood-2013-05-503177 PMC3811179

[13] Himburg HA, Muramoto GG, Daher P, Meadows SK, Russell JL, Doan P, Pleiotrophin regulates the expansion and regeneration of hematopoietic stem cells. Nat Med. 2010;16(4):475–82. 20305662 10.1038/nm.2119 PMC3689427

[14] Boitano AE, Wang J, Romeo R, Bouchez LC, Parker AE, Sutton SE, Aryl hydrocarbon receptor antagonists promote the expansion of human hematopoietic stem cells. Science. 2010;329(5997):1345–8. 20688981 10.1126/science.1191536 PMC3033342

[15] Chen X, Skutt-Kakaria K, Davison J, Ou YL, Choi E, Malik P, G9a/GLP-dependent histone H3K9me2 patterning during human hematopoietic stem cell lineage commitment. Genes Dev. 2012;26(22):2499–511. 23105005 10.1101/gad.200329.112 PMC3505820

[16] Fares I, Chagraoui J, Gareau Y, Gingras S, Ruel R, Mayotte N, Cord blood expansion. Pyrimidoindole derivatives are agonists of human hematopoietic stem cell self-renewal. Science. 2014;345(6203):1509–12. 25237102 10.1126/science.1256337 PMC4372335

[17] Peled T, Mandel J, Goudsmid RN, Landor C, Hasson N, Harati D, Pre-clinical development of cord blood-derived progenitor cell graft expanded ex vivo with cytokines and the polyamine copper chelator tetraethylenepentamine. Cytotherapy. 2004;6(4):344–55. 16146887 10.1080/14653240410004916

[18] de Lima M, McMannis J, Gee A, Komanduri K, Couriel D, Andersson BS, Transplantation of ex vivo expanded cord blood cells using the copper chelator tetraethylenepentamine: a phase I/II clinical trial. Bone Marrow Transplant. 2008;41(9):771–8. 18209724 10.1038/sj.bmt.1705979 PMC4086223

[19] Tatetsu H, Armant M, Wang F, Gao C, Ueno S, Tian X, Maintenance and enhancement of human peripheral blood mobilized stem/progenitor cell engraftment after ex vivo culture via an HDACi/SALL4 axis (3465). Exp Hematol. 2019;75:53–63 e11. 31260717 10.1016/j.exphem.2019.06.473 PMC6719293

[20] Kohlhase J, Schuh R, Dowe G, Kuhnlein RP, Jackle H, Schroeder B, Isolation, characterization, and organ-specific expression of two novel human zinc finger genes related to the Drosophila gene spalt. Genomics. 1996;38(3):291–8. 8975705 10.1006/geno.1996.0631

[21] Frei E, Schuh R, Baumgartner S, Burri M, Noll M, Jurgens G, Molecular characterization of spalt, a homeotic gene required for head and tail development in the Drosophila embryo. EMBO J. 1988;7(1):197–204. 16453821 10.1002/j.1460-2075.1988.tb02800.x PMC454251

[22] Kuhnlein RP, Frommer G, Friedrich M, Gonzalez-Gaitan M, Weber A, Wagner-Bernholz JF, spalt encodes an evolutionarily conserved zinc finger protein of novel structure which provides homeotic gene function in the head and tail region of the Drosophila embryo. EMBO J. 1994;13(1):168–79. 7905822 10.1002/j.1460-2075.1994.tb06246.x PMC394790

[23] Tatetsu H, Kong NR, Chong G, Amabile G, Tenen DG, and Chai L. SALL4, the missing link between stem cells, development and cancer. Gene. 2016;584(2):111–9. 26892498 10.1016/j.gene.2016.02.019 PMC4823161

[24] Eildermann K, Aeckerle N, Debowski K, Godmann M, Christiansen H, Heistermann M, Developmental expression of the pluripotency factor sal-like protein 4 in the monkey, human and mouse testis: restriction to premeiotic germ cells. Cells Tissues Organs. 2012;196(3):206–20. 22572102 10.1159/000335031

[25] Miettinen M, Wang Z, McCue PA, Sarlomo-Rikala M, Rys J, Biernat W, SALL4 expression in germ cell and non-germ cell tumors: a systematic immunohistochemical study of 3215 cases. Am J Surg Pathol. 2014;38(3):410–20. 24525512 10.1097/PAS.0000000000000116 PMC4041084

[26] Cao D, Guo S, Allan RW, Molberg KH, and Peng Y. SALL4 is a novel sensitive and specific marker of ovarian primitive germ cell tumors and is particularly useful in distinguishing yolk sac tumor from clear cell carcinoma. Am J Surg Pathol. 2009;33(6):894–904. 19295406 10.1097/PAS.0b013e318198177d

[27] Kohlhase J, Heinrich M, Schubert L, Liebers M, Kispert A, Laccone F, Okihiro syndrome is caused by SALL4 mutations. Hum Mol Genet. 2002;11(23):2979–87. 12393809 10.1093/hmg/11.23.2979

[28] Gao C, Kong NR, Li A, Tatetu H, Ueno S, Yang Y, SALL4 is a key transcription regulator in normal human hematopoiesis. Transfusion. 2013;53(5):1037–49. 22934838 10.1111/j.1537-2995.2012.03888.x PMC3653586

[29] Shen B, Zhang Y, Dai W, Ma Y, and Jiang Y. Ex-vivo expansion of nonhuman primate CD34(+) cells by stem cell factor Sall4B. Stem Cell Res Ther. 2016;7(1):152. 27765075 10.1186/s13287-016-0413-1 PMC5072326

[30] Mossahebi-Mohammadi M, Atashi A, Kaviani S, and Soleimani M. Efficient Expansion of SALL4-Transduced Umbilical Cord Blood Derived CD133+Hematopoietic Stem Cells. Acta Med Iran. 2017;55(5):290–6. 28724268

[31] Milanovich S, Peterson J, Allred J, Stelloh C, Rajasekaran K, Fisher J, Sall4 overexpression blocks murine hematopoiesis in a dose-dependent manner. Exp Hematol. 2015;43(1):53–64 e1-8. 25246269 10.1016/j.exphem.2014.09.004 PMC4268405

[32] Gao C, Dimitrov T, Yong KJ, Tatetsu H, Jeong HW, Luo HR, Targeting transcription factor SALL4 in acute myeloid leukemia by interrupting its interaction with an epigenetic complex. Blood. 2013;121(8):1413–21. 23287862 10.1182/blood-2012-04-424275 PMC3578956

[33] Liu BH, Jobichen C, Chia CSB, Chan THM, Tang JP, Chung TXY, Targeting cancer addiction for SALL4 by shifting its transcriptome with a pharmacologic peptide. Proc Natl Acad Sci U S A. 2018;115(30):E7119–E28. 29976840 10.1073/pnas.1801253115 PMC6065023

[34] Lu J, Jeong HW, Kong N, Yang Y, Carroll J, Luo HR, Stem cell factor SALL4 represses the transcriptions of PTEN and SALL1 through an epigenetic repressor complex. PLoS One. 2009:4(5):e5577. 19440552 10.1371/journal.pone.0005577 PMC2679146

[35] Yong KJ, Gao C, Lim JS, Yan B, Yang H, Dimitrov T, Oncofetal gene SALL4 in aggressive hepatocellular carcinoma. N Engl J Med. 2013;368(24):2266–76. 23758232 10.1056/NEJMoa1300297 PMC3781214

[36] Li A, Yang Y, Gao C, Lu J, Jeong HW, Liu BH, A SALL4/MLL/HOXA9 pathway in murine and human myeloid leukemogenesis. J Clin Invest. 2013;123(10):4195–207. 24051379 10.1172/JCI62891 PMC3784519

